# The Impact of Stressful Life Events on Excessive Alcohol Consumption in the French Population: Findings from the GAZEL Cohort Study

**DOI:** 10.1371/journal.pone.0087653

**Published:** 2014-01-27

**Authors:** Sara L. Tamers, Cassandra Okechukwu, Alex A. Bohl, Alice Guéguen, Marcel Goldberg, Marie Zins

**Affiliations:** 1 Department of Social and Behavioral Sciences, Harvard School of Public Health, Boston, Massachusetts, United States of America; 2 Center for Community-Based Research, Dana-Farber Cancer Institute, Boston, Massachusetts, United States of America; 3 Mathematica Policy Research, Cambridge, Massachusetts, United States of America; 4 INSERM, UMRS 1018, Centre for Research in Epidemiology and Population Health, Population-based Cohorts Research Platform, Villejuif, France; 5 Université de Versailles Saint-Quentin, Versailles, France; University of Granada, Spain

## Abstract

**Background:**

Major life changes may play a causative role in health through lifestyle factors, such as alcohol. The objective was to examine the impact of stressful life events on heavy alcohol consumption among French adults.

**Methods:**

Trajectories of excessive alcohol consumption in 20,625 employees of the French national gas and electricity company for up to 5 years before and 5 years after an event, with annual measurements from 1992. We used repeated measures analysis of time series data indexed to events, employing generalized estimating equations.

**Results:**

For women, excessive alcohol use increased before important purchase (p = 0.021), children leaving home (p<0.001), and death of loved ones (p = 0.03), and decreased before widowhood (p = 0.015); in the year straddling the event, increased consumption was observed for important purchase (p = 0.018) and retirement (p = 0.002); at the time of the event, consumption decreased for marriage (p = 0.002), divorce, widowhood, and death of loved one (all p<0.001), and increased for retirement (p = 0.035). For men, heavy alcohol consumption increased in the years up to and surrounding the death of loved ones, retirement, and important purchase (all p<0.001), and decreased after (all p<0.001, except death of loved one: p = 0.006); at the time of the event, consumption decreased for all events except for children leaving home and retirement, where we observed an increase (all p<0.001). For women and men, heavy alcohol consumption decreased prior to marriage and divorce and increased after (all p<0.001, except for women and marriage: p = 0.01).

**Conclusion:**

Stressful life events promote healthy and unhealthy alcohol consumption. Certain events impact alcohol intake temporarily while others have longer-term implications. Research should disentangle women's and men's distinct perceptions of events over time.

## Introduction

Stressful life events (SLEs) leading to or requiring a life adjustment can have a significant impact on health [1], [2], [3], [4], [5], [6], [7], [8]. Defined as events that significantly disturb daily routines [9]—as opposed to minor stressors—SLEs can include both desirable events (e.g., marriage), undesirable events (e.g., death of loved ones), as well as mixed ones that comprise both desirable and undesirable elements (e.g., retirement) [10]. As a result of stress, certain SLEs can lead to the uptake of unhealthy behaviors, such as excessive alcohol intake [11]—a chief cause of morbidity [12] and early mortality [13]; conversely, others may contribute to healthy behaviors [14]. An increasing number of studies have examined the complex relationship between SLEs and alcohol [15], [16], [17], [18], [19], [20]; however, methodological differences restrict the extent of their application to public health interventionists and policy makers.

Relatively few studies have explored the relationships between SLEs and alcohol, longitudinally [14], [15], [21], [22], [23], which curbs the ability to understand temporality and trajectories in alcohol use. Of those studies that have taken a longitudinal approach, many have grouped SLEs rather than assess the individual impact of a number of these key changes [22]. Given that events may be perceived as desirable, undesirable, or mixed, this method is limited as it does not capture the fact that each SLE may influence health behaviors differently. Additionally, much of the research on this theme has focused on younger generations, despite the fact that middle-aged and older adult populations have become an increasingly prominent group. As such, they are overwhelmingly affected by important SLEs, such as children leaving home and widowhood; however, comparatively little is known about the natural history of alcohol use or misuse among these groups [15], [24], [25] and of its association with SLEs experienced across a broad timeframe.

Finally, a vast majority of alcohol research [26] and studies on its relationship with SLEs stem from the U.S. [15], [17], [21], [22]. Exploring these associations in non-U.S. contexts is important to further the field, in view of the vast social and cultural differences in both the aging process and alcohol use in European contexts. France is a unique country to study alcohol trajectories and their relationship with SLEs, as the French have one of the world's longest life expectancies while also having one of the highest alcohol consumption rates among industrialized nations [27]. Indeed, alcohol is often consumed daily by a majority of the French population [28], with only 35 percent of women and 17 percent of men abstaining from alcohol [28]. More recently, excessive drinking has become a public health issue in France, resulting in premature deaths [28], [29].

Building upon earlier work [27], [30], [31], the goal of this study was to examine the impact of SLEs representing two major life domains (i.e., interpersonal and financial) on excessive alcohol intake among a group of middle-aged and older French adults.

## Methods

### Study population

We used data from the GAZEL cohort consisting of 20,625 employees, aged 35–50 years at inception, of the French national gas and electricity company who were recruited to complete an annual self-administered questionnaire since 1989. Participants were asked a variety of questions related to their socio-demographics, health behaviors, life experiences, and occupational characteristics. The GAZEL cohort has been described in great detail in previous studies [32], [33].

#### Ethics statement

Approval for the GAZEL study was received by La Commission Nationale de l'Informatique et des Libertés and from L'Institut National de la Santé et de la Recherche Médicale's Instutional Review Board.

### Measures

#### Stressful life events

Guided by previous literature, we included eight SLEs that capture common experiences in interpersonal and financial-related domains [15], [27], [30], [34]; interpersonal: 1) marriage, 2) children leaving home, 3) divorce, 4) widowhood, and 5) death of loved ones; financial: 6) employment promotion, 7) important purchases, and 8) retirement. All SLEs were ranked in Holmes' and Rahe's Social Readjustment Scale as significant life events that pertain to major areas impacting adult life [10]. We determined the first instance after 1989 that an individual experienced a given SLE in the past 12 months. SLEs were coded as dichotomous (yes/no) variables.

#### Excessive alcohol consumption

Given that heavy drinking has become an important public health concern among many industrialized nations, including France [28], [29], we focused on excessive alcohol intake as our outcome. GAZEL participants' alcohol consumption was ascertained from a validated instrument, culturally-adapted to the drinking habits of populations who consume alcohol on a usual basis, such as the French and Italians [31], [35]. Questions included: *“Have you consumed any wine, beer or cider, spirits over the past week? If yes, what was the maximum quantity per day (number of glasses)? On how many days during the past week did you drink wine, beer or cider, and spirits?”* To facilitate participant response, drawings representing standard glasses for each alcohol type and amount were included below each question. Weekly maximum alcohol consumption was computed for wine, beer or cider, and spirits by multiplying the maximum number of glasses consumed by the number of days of drinking. As per the World Health Organization, the U.S. National Institute on Alcohol Abuse and Alcoholism, and France's Institut de Recherche et Documentation en Economie de la Santé guidelines, this was defined as at least 14 drinks per week for women and at least 28 drinks per week for men [28], [36], [37].

#### Covariates

Research has shown that certain socio-demographic variables, including age (35–39, 40–44, 45–49, 50–54, 55–59, 60–64, 65+) [18], [20], health status (excellent, good, fair, poor) [38], education (< high school, high school, > high school) [18], [20], and employment grade (manual worker/clerk, administrative associate/technician, manager) [31] may account for some of the observed effect of SLEs on alcohol. These covariates were included a priori and adjusted for in our multivariable models as they were found to be statistically significant at the bivariate stage.

### Statistical analyses

The GAZEL study began collecting participant data in 1989; however, alcohol data were only collected from 1992 onwards. At the time this study was performed, the authors had access to survey data through the end of 2010. We included solely those individuals who provided information on alcohol consumption for the year of the SLE, as well as for at least two surveys before and two surveys after each SLE. Thus, the final analyses used up to 11 years of data for each individual who experienced an event. Individuals could experience multiple events within and between categories. Evidence has shown that first occurrences are more impactful than subsequent ones as the latter often become progressively less critical [39]; thus, our analysis focused on the first time an event was experienced in a given category for each individual. Women and men typically have different drinking habits [28] and may respond differently to SLEs [18], [40]. Further, evidence has shown that the maginitude of the effect between SLEs and alcohol behavior may be different by sex [18], [20]; since effect modification was found, we present stratified analyses.

The statistical analyses had two purposes. First, we sought to test for differences in heavy alcohol consumption in four different time periods (before, during, after, and at the time of each event), adjusting for socio-demographics and temporal trends in alcohol consumption. To test for heavy alcohol consumption *before* the experienced event—considered as time 0—we tested for a difference one year pre-event compared to five years pre-event (year −1 versus −5). To test for heavy alcohol consumption *during* the time of the event, we tested for a difference one year post-event compared to one year pre-event (year +1 versus −1). To test for heavy alcohol consumption *after* the event, we tested for a difference five years post-event compared to one year post-event (year +5 versus +1). Finally, we examined heavy alcohol consumption *at* the time of the event (year 0).

Second, we plotted model-predicted excessive alcohol intake for five years before and five years after each SLE, adjusted for covariates. Least-squares means estimates were used to produce graphs showing trajectories in heavy alcohol consumption rates over time.

The data were correlated by individuals; thus, we used repeated measures analysis of time series data indexed to life events. To do so, we used logistic regression with generalized estimating equations (GEE) and a logit link and binomial family, in STATA version 10 [41]. To allow for time varying covariates, we used an independent working correlation with robust standard errors. The unit of analysis was the person-year, and each person contributed at least five but up to 11 observations. Such a model has been used previously for studies using GAZEL data [42], [43].

## Results

Over 17 years of observation (1992 through 2008), 98% (20,224 of 20,625) of the GAZEL cohort participants experienced at least one SLE. Missing survey response rates varied by SLE and survey period relative to the event, but the majority of participants (80–95%) had observations in pre- and post-event survey periods. Depending on the event and sex, between 577 (women who got married) to 14,442 (men who retired) individuals experienced an event; thus, the final sample size varied for each event and outcome.

Tables 1 and 2 describe the percentage of participants who experienced SLEs, separately for women and men, within socio-demographic categories. For both sexes, the most frequently experienced event was retirement; the majority of SLEs occurred before the age of 60; and widowhood occurred at older ages. Across all events, roughly 60% of women and 70% of men had greater than a high school education, and the majority reported being in excellent or good health, at the time of the event. Most women and men were employed in an intermediate position (defined as administrative associate/technician) as of 1990.

**Table 1:** Socio-demographic characteristics for GAZEL women, at time of each life event.

	Marriage	Employment promotion	Important purchase	Children leaving home	Divorce	Retirement	Widowhood	Death of a loved one
								
**N**	**577**	**829**	**3237**	**2417**	**987**	**4465**	**589**	**4147**
**Age at event**								
35–39	24%	10%	3%	0%	24%	0%	10%	3%
40–44	37%	37%	17%	8%	33%	0%	15%	20%
45–49	22%	33%	25%	35%	17%	0%	16%	27%
50–54	9%	17%	25%	36%	11%	33%	14%	27%
55–59	5%	3%	19%	16%	9%	53%	16%	16%
60–64	2%	0%	9%	4%	5%	14%	20%	7%
65+	1%	0%	2%	1%	1%	0%	9%	2%
**Education**								
<High school	27%	26%	26%	24%	26%	29%	29%	26%
High school	11%	15%	12%	12%	13%	9%	9%	11%
> High school	60%	56%	60%	62%	58%	60%	59%	60%
**Self-rated health**								
Excellent	28%	21%	24%	21%	29%	17%	24%	23%
Good	44%	40%	53%	53%	41%	38%	45%	53%
Fair	12%	11%	19%	22%	18%	12%	22%	21%
Poor	1%	1%	2%	2%	3%	1%	3%	2%
**Employment**								
Manual Worker/Clerk	29%	63%	21%	22%	26%	22%	23%	22%
Admin Associate/Technician	64%	37%	69%	68%	66%	70%	70%	69%
Manager	8%	0%	10%	10%	8%	8%	7%	9%

Note: Cells may not always add up to 100% or total N due to rounding and/or missing data

**Table 2:** Socio-demographic characteristics for GAZEL men, at time of each life event

	Marriage	Employment promotion	Important purchase	Children leaving home	Divorce	Retirement	Widowhood	Death of a loved one
								
**N**	**738**	**1518**	**10012**	**6449**	**1845**	**14442**	**1126**	**11459**
**Age at event**								
35–39	0%	0%	0%	0%	0%	0%	0%	0%
40–44	35%	15%	4%	0%	30%	0%	7%	5%
45–49	38%	49%	26%	18%	27%	0%	12%	26%
50–54	17%	32%	33%	43%	15%	34%	14%	33%
55–59	7%	3%	24%	29%	16%	55%	25%	22%
60–64	2%	0%	11%	8%	10%	10%	30%	11%
65+	1%	0%	3%	1%	2%	0%	12%	3%
**Education**								
<High school	20%	19%	18%	16%	19%	19%	17%	19%
High school	8%	8%	6%	6%	7%	6%	7%	6%
> High school	70%	72%	74%	76%	72%	73%	73%	74%
**Self-rated health**								
Excellent	35%	26%	31%	29%	32%	23%	26%	30%
Good	43%	43%	53%	52%	43%	38%	52%	52%
Fair	10%	11%	14%	17%	16%	11%	17%	15%
Poor	2%	1%	1%	1%	2%	1%	1%	1%
**Employment**								
Manual Worker/Clerk	14%	29%	10%	10%	13%	13%	11%	11%
Admin Associate/Technician	57%	71%	53%	53%	56%	56%	53%	55%
Manager	30%	0%	36%	38%	31%	32%	35%	34%

Note: Cells may not always add up to 100% or total N due to rounding and/or missing data

Tables 3 and 4 show odds of heavy alcohol consumption in four different time periods (years −1 versus −5; +1 versus −1; +5 versus +1; year 0) for each SLE and for women and men, separately. Most of the significant results observed for both sexes were increases in heavy alcohol use, especially during the time period surrounding the event (men). For women, excessive alcohol use increased in the years before important purchase, children leaving home, and death of loved one, and decreased for widowhood. In the year straddling the event, increased consumption was observed for important purchase and retirement, only. At the time of the event, excessive alcohol use decreased for marriage, divorce, widowhood, and death of loved one, and increased for retirement. For men, heavy alcohol consumption increased in the years up to and surrounding the death of a loved one, retirement, and important purchase, and then decreased after the experienced event. Increased consumption was observed in the years before children leaving home, and in the year surrounding and after receiving an employment promotion. At the time of the event, excessive alcohol use decreased for all events except for children leaving home and retirement, where we observed an increase.

**Table 3:** Heavy alcohol use for women during and around stressful life events*.

		Year −1 vs. −5			Year +1 vs. −1			Year +5 vs. +1			Year 0		
		*OR*	*P*	*95% CI*	*OR*	*P*	*95% CI*	*OR*	*P*	*95% CI*	*OR*	*P*	*95% CI*
Marriage		0.192	**0.028**	0.04–0.84	1.7	0.568	0.27–10.71	5.35	**0.01**	1.49–19.24	0.24	**0.002**	0.1–0.59
Employment promotion		0.991	0.99	0.25–3.86	2.06	0.073	0.94–4.54	1.41	0.152	0.88–2.29	0.6	0.09	0.33–1.07
Important purchase		1.4	**0.021**	1.05–1.86	1.26	**0.018**	1.04–1.55	0.854	0.135	0.7–1.05	0.87	0.075	0.74–1.01
Children leaving home		1.9	**<0.001**	1.48–2.64	1.09	0.37	0.9–1.34	0.958	0.717	0.76–1.21	0.91	0.31	0.75–1.1
Divorce		0.15	**<0.001**	0.07–0.33	1.47	0.278	0.73–2.99	3.16	**<0.001**	1.77–5.68	0.22	**<0.001**	0.14–0.36
Retirement		1.23	0.063	0.99–1.54	1.34	**0.002**	1.11–1.62	0.877	0.187	0.72–1.07	1.19	**0.035**	1.01–1.4
Widowhood		0.516	**0.015**	0.3–0.88	0.857	0.453	0.58–1.28	1.28	0.356	0.75–2.21	0.4	**<0.001**	0.24–0.64
Death of loved one		1.33	**0.03**	1.03–1.74	1.11	0.245	0.93–1.35	1.04	0.607	0.88–1.25	0.76	**<0.001**	0.66–0.88

Year 0 is the year of the experienced life event

* Adjusted for age, self-rated health, and education and employment grade at time of event

**Table 4:** Heavy alcohol use for men during and around stressful life events*.

		Year −1 vs. −5			Year +1 vs. −1			Year +5 vs. +1			Year 0		
		*OR*	*P*	*95% CI*	*OR*	*P*	*95% CI*	*OR*	*P*	*95% CI*	*OR*	*P*	*95% CI*
Marriage		0.101	**<0.001**	0.04–0.24	2.45	**0.015**	1.19–5.05	4.41	**<0.001**	2.79–6.99	0.14	**<0.001**	0.09–0.23
Employment promotion		1.08	0.7	0.72–1.64	1.74	**<0.001**	1.37–2.21	1.26	**0.014**	1.05–1.53	0.69	**<0.001**	0.56–0.84
Important purchase		1.36	**<0.001**	1.25–1.49	1.32	**<0.001**	1.25–1.41	0.885	**<0.001**	0.83–0.94	0.9	**<0.001**	0.85–0.95
Children leaving home		2.27	**<0.001**	2.06–2.52	0.989	0.769	0.93–1.06	0.938	0.086	0.87–1.01	1.3	**<0.001**	1.22–1.39
Divorce		0.379	**<0.001**	0.3–0.48	1.28	**0.009**	1.06–1.55	2	**<0.001**	1.66–2.42	0.39	**<0.001**	0.33–0.46
Retirement		1.15	**<0.001**	1.08–1.23	1.38	**<0.001**	1.32–1.47	0.869	**<0.001**	0.83–0.92	1.21	**<0.001**	1.15–1.27
Widowhood		0.845	0.141	0.68–1.06	1.1	0.251	0.93–1.31	1	0.976	0.8–1.26	0.71	**<0.001**	0.6–0.83
Death of loved one		1.27	**<0.001**	1.17–1.38	1.36	**<0.001**	1.29–1.45	0.925	**0.006**	0.88–0.98	0.86	**<0.001**	0.81–0.9

Year 0 is the year of the experienced life event.

* Adjusted for age, self-rated health, and education and employment grade at time of event.

For both sexes, marriage and divorce followed similar patterns in that heavy alcohol intake initially decreased and then increased in the years after the event. Of note, men increased their intake around the time of the event and in the period after, while women only increased theirs in the years after the event. Further supplemental analyses of risk of excessive alcohol use for additional time periods (Tables S1 and S2 in File S1) are included as a supplement to the paper.

**Figure 1:**
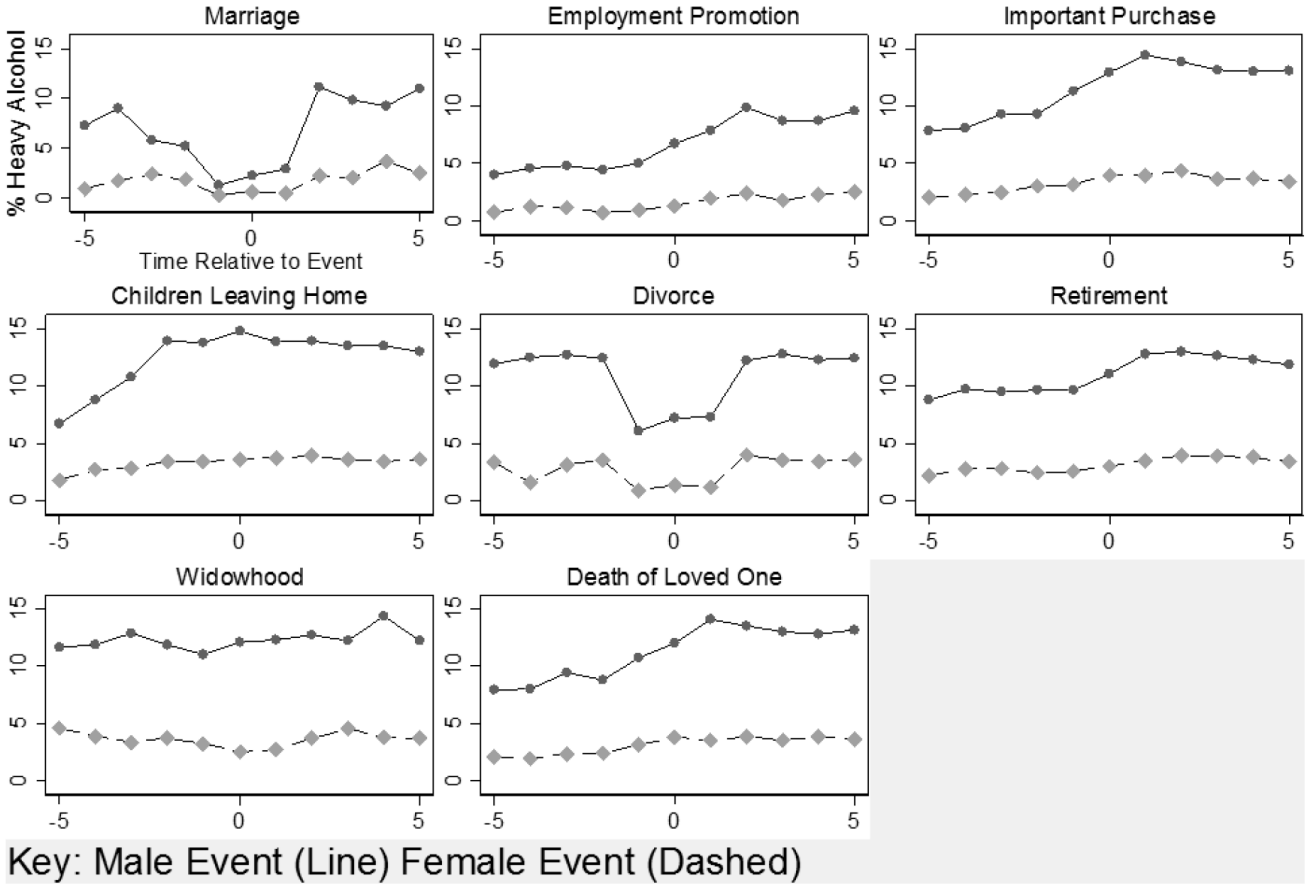
Percentage of heavy drinkers around time of stressful life events, separately for women and men.

Figure 1 plots model-predicted heavy alcohol consumption in the five years before and five years after each experienced SLE, adjusted for covariates. These graphs provide perspective to the results provided in tables 3 and 4, as they illustrate trajectories of excessive alcohol consumption relative to a life event. In all cases, heavy consumption was more frequent for men. As seen in Figure 1, at the time of marriage and divorce, we noted the most significant decrease in the percentage of heavy alcohol consumers. For all other events, we observed a gradual increase over time.

## Discussion

### Summary of findings

In this study, we sought to examine the relationship between a number of commonplace SLEs capturing important life domains and excessive alcohol consumption before, during, and after the experienced event, among a large sample of French women and men. We also further explored trends of heavy alcohol rates over time for each SLE. With some exceptions, overall, within each sample of women and men, our findings suggest a differential impact by time period, domain, and SLE type; thus, some generalizations can be made.

In the years prior to experiencing an event, for both women and men, we perceived decreases in heavy alcohol consumption before marriage, divorce, and widowhood (women only), and increases before important purchase, children leaving home, retirement (men only), and death of loved ones. These fluxes in heavy alcohol use in the years prior to experiencing an event may be due to the anticipation of said events; evidence has shown that anticipated or lead effects may be reflected months or even years before the event occurs [44]. In the year before compared to the year after an experienced event, we noted only increases in excessive alcohol intake, with women experiencing these for important purchase and retirement, and men experiencing these for all SLEs except for children leaving home and widowhood. In the years subsequent to experiencing an event, we saw an increase in heavy alcohol consumption for women after marriage and divorce. For men, we also found an increase in the years after marriage and divorce, as well as after employment promotion, and decreases after important purchase, retirement, and death of loved one. These findings suggest that certain SLEs may impact heavy alcohol use even years after the event [45]. At the time of the event, we observed mostly decreased heavy alcohol use for over half of the examined events for women and all events for men.

### Comparison with previous studies

Though some disparities were noted, by and large, our results are in accordance with previous research on similar themes revealing that many SLEs have an impact on alcohol behavior not only at the time of the event, as supported by our findings, but also in the years before and after [16], [21], [22], [23], [31], [46]. Like Perreira and Sloan (2001), we found that retirement was associated with increases in intake and that marriage and divorce were associated with both increases and decreases in heavy alcohol use at different time periods [22]. As reflected in prior evidence, we found increases in alcohol consumption in the years precipitating children leaving home [47], [48]. Previous studies have found that experiencing the death of a loved one and widowhood can have varying impacts on drinking practices [22], [49]. Both involve loss; still, similar alcohol behavior patterns are not assured for both, across time, or by sex, which could help explain differences in the directionality of our findings. For instance, an anticipated or unexpected death might influence health outcomes differently [50], and the degree of care-giving in the case of an anticipated bereavement [51] might also play a role. The nature and saliency of the relationship between the bereaved and deceased could have a variable influence on alcohol intake [49], [52], as could a change in social network norms [53]. We found no significant results with respect to heavy alcohol use for women receiving an employment promotion in our main analyses but significant ones for men, which is in line with other like studies [54]; however, the directionality for men was opposite. Making a large purchase, which could be viewed as a proxy measure for reduced finances or more financial strain, has been associated with increased alcohol use among older adults [55].

While we cannot compare differences between the two separate samples of women and men, we can comment on perceived trends across both. Men's excessive alcohol consumption tended to be more frequently impacted by life events, which has been observed previously [18], [23]. This finding may be explained in part by the fact that there are sex differences in both alcohol consumption [56] and in the impact of life events on intake; posited explanations include distinct support seeking and coping styles [57], as well as stigmatization and societal norms [18]. We also observed that women typically experienced increases in their heavy alcohol intake across most of the time periods, whereas men appeared to experience both increases and decreases in intake, more equally. Financial-related SLEs seemed to influence men's heavy alcohol use more often compared to women, who were more frequently impacted by interpersonal events. Still, we observed similar patterns for interpersonal events for both sexes, which may suggest that a change in social relationships has a greater impact on heavy alcohol use.

When examining overall differences across time, certain SLEs influenced excessive alcohol intake years after the event, while others appeared to have only short-term effects or none at all. The latter may be a result of adaptation [45], meaning that individuals tend to adjust relatively quickly to life changes [58] because they find explanations for undesirable events [59] and get used to desirable ones [45]. This may partially explain some perceived trends in the years subsequent to experiencing certain SLEs, intimating that levels of heavy alcohol use eventually return to those similar pre-event. It may also provide clarity for some seemingly counter-intuitive results—marital shift resulting in a desirable outcome (i.e., marriage) and undesirable one (i.e., divorce)—showing similar alcohol use patterns. Indeed, we found that a number of SLEs—be they desirable, undesirable, or mixed—had both a healthy and unhealthy impact on heavy alcohol intake in different time periods. Research has found that SLEs perceived to be positive (i.e., marriage, employment promotion), negative (i.e., divorce, widowhood, death of loved one), or mixed (i.e., important purchase, children leaving home, retirement) may have distinct associations with well-being measures [45] and that this impact may even prove to be differential by SLE, across time. Further, the impact of negatively-perceived SLEs may be stronger and/or more persistent than positively- or mixed-perceived ones [60], [61], [62].

### Limitations and strengths

One potential limitation of this study is recall bias given that respondents were asked about events experienced in the previous 12 months. It has also been suggested that certain SLEs may be subject to reporting bias as those perceived to be negative may be more likely to be recollected [16], [63]. Overestimation of the proportion of excessive drinkers could be present if levels of drinking vary greatly day-to-day; however, this would be balanced by the fact that alcohol intake is typically underreported [31]. Even so, the way weekly intake was computed should not contribute much to overestimation; this is further strengthened by the fact that the rate of heavy drinkers in the GAZEL cohort is comparable to that in the French population measured via other surveying tools [64]. There likely exist cultural differences that may limit the generalizability of our findings to solely French or Mediterranean populations with similar alcohol drinking practices. Still, this study has important strengths. We incorporated a number of SLEs that are universal experiences covering central life domains. We also studied heavy alcohol use, which has become an increasingly common public health issue among industrialized nations. An additional strength of our study is that the GAZEL cohort enabled us to look at trajectories within a large and stable population over a number of years. Moreover, few large-scale epidemiological studies have explored these relationships longitudinally and among both a middle-aged and/or older-adult population in a non-US context [14], [21], [22], [23].

### Conclusion

In summary, our findings suggest that a number of SLEs across significant domains may promote both healthy and unhealthy alcohol consumption at different periods in time. While some events might impact alcohol intake only temporarily, others may have longer-term implications. What’s more, even if a short-term effect is not detected, experiencing a SLE might set an individual on a trajectory of increased heavy alcohol consumption over time. From a clinical practice stand-point, providers should regularly ascertain middle-aged and older adults' drinking habits—paying particular focus to experienced or imminent SLEs—and offer appropriate resources to address unhealthy drinking practices, should they exist. From a research perspective, given observed differences by domain, SLE type, and timeframe, future studies should attempt to disentangle distinct perceptions of SLEs (i.e., desirable, undesirable, or mixed) over time. Such evidence could help elucidate the mechanisms by which SLEs can both promote deleterious drinking practices and encourage healthier ones.

## Supporting Information

File S1 **Tables S1 and S2.** Table S1. Logistic regression predicting heavy alcohol use around stressful life events for women in the GAZEL cohort. Table S2. Logistic regression predicting heavy alcohol use around stressful life events for men in the GAZEL cohort.
(DOCX)
