# Supplementary material for: The Impact of Stressful Life Events on Excessive Alcohol Consumption in the French Population: Findings from the GAZEL Cohort Study
Source: PLoS One. 2014 Jan 27;9(1):e87653. doi: 10.1371/journal.pone.0087653 (PMC3903768; doi:10.1371/journal.pone.0087653)
Supplement: File S1 — Tables S1 and S2. Table S1. Logistic regression predicting heavy alcohol use around stressful life events for women in the GAZEL cohort. Table S2. Logistic regression predicting heavy alcohol use around stressful life events for men in the GAZEL cohort. (DOCX) [file pone.0087653.s001.docx]

**Supporting Information**

To supplement results presented in the paper, we conducted additional analyses for 3 other time periods. Tables S1 and S2 show odds of heavy alcohol consumption (years +5 vs. -5, +1 vs. -5, +5 vs. -1) for each SLE and for women and men, separately.

For women, comparing five years after the event to five years before, there is a higher risk of excessive alcohol use for important purchase, children leaving home, retirement, and death of loved one. Comparing one year after the event to five years before, excessive alcohol use increased for important purchase, children leaving home, retirement, and death of loved one, and decreased for divorce and widowhood. Comparing five years after the event to one year before, excessive alcohol use increased for marriage, employment promotion, and divorce.

For men, comparing five years after the event to five years before, there is higher risk of excessive alcohol use for employment promotion, important purchase, children leaving home, retirement, and death of loved one. Comparing one year after the event to five years before, excessive alcohol use increased for employment promotion, important purchase, children leaving home, retirement, and death of loved one, and decreased for marriage and divorce. Comparing five years after the event to one year before, excessive alcohol use increased for marriage, employment promotion, important purchase, divorce, retirement, and death of loved one.
